# Does Alice Live Here Anymore? Autonomy and Identity in Persons Living and Dying With Dementia

**DOI:** 10.3389/fpsyt.2021.700567

**Published:** 2021-07-22

**Authors:** Migita M. D'cruz

**Affiliations:** Geriatric Psychiatry Unit, Department of Psychiatry, National Institute of Mental Health and Neuro Sciences, Bangalore, India

**Keywords:** dementia, neurocognitive disorders, euthanasia, physician assisted dying, physician assisted death, decision making capacity, legal competence, consent and refusal to treatment

## Abstract

Conventional scientific definitions of dementia, or its newer proposed alternate—neurocognitive disorders place emphasis upon cognitive function, particularly memory. The changes in thought, emotion, behavior, personality, and biological function are usually considered only of secondary importance. At the core of the illness, however, lies a progressive loss of self, and by extension, of personhood, identity, autonomy, and agency. The identity of the person living with dementia, and the deterioration of a sense of self assumes significance in planning end of life care, including palliative care. A consideration of self and identity is also significant where physician assisted death, incorporating euthanasia, has legal sanctity. As dementia progresses, there is usually a progressive loss of personal decision making capacity and legal competence. Shared decision making, advance care directives and proxy representatives are options available to safeguard autonomy and agency in such cases. Advance care directives are often treated as static documents. The loss of self and deterioration of identity in persons with dementia means, that there is a psychological discontinuity across time and space, though biological continuity is retained. The discontinuity in self and identity however, imply that the person with dementia changes considerably and so too may values and beliefs. A document which best reflected the wishes of the person with dementia in the past, may not always do so now. Advance directives and proxy representatives may need to be dynamic and evolve over time, particularly where end of life care and physician assisted death is being invoked.

## Background—Dementia and Identity

Dementia has been classically defined, by Jeffrey L. Cummings, in 1992, is as an acquired syndrome of intellectual impairment produced by brain dysfunction, usually organic. (1) A more elaborate, alternative definition of the condition, by the ICD-10 Classification of Mental and Behavioral Disorders (ICD-10), also offered in 1992, is that of a syndrome due to disease of the brain, usually of a chronic or progressive nature, in which there is disturbance of multiple higher cortical functions, including memory, thinking, orientation, comprehension, calculation, learning capacity, language and judgement (2).

These definitions, while useful in the clinical, academic and research context, however, run the risk of a narrow and excessively cognitive function based view of the omnibus of diseases that compose dementing illnesses. (3) Further, the word dementia, in common and general public usage, is often understood as a loss of one specific cognitive function—memory. It is thus considered synonymous by implication, and often indistinguishable from amnesia or a loss of memory. Indeed the very term, dementia, derives from the Latin root word *demens*, implying mad, raving, or out of one's mind. (4, 5) The newer term—neurocognitive disorders, proposed by the Diagnostic and Statistical Manual of Mental Disorders, in its 5th edition (DSM-5) in 2013, while broadening the scope of the definition with emphasis upon cognitive functions other than memory, continues to limit itself primarily to cognition (6).

A criticism of this approach to dementia, articulated by Lawrence Cohen, among others, is that it fails to highlight adequately the changes in perception, thought, emotion, behavior, and biological functions that accompany cognitive impairment in persons with dementia. (7) These are considered of secondary importance and lumped together under the rubric of the behavioral and psychological symptoms associated with dementia (BPSD). Yet, the BPSD occur in 50% of persons diagnosed with dementia within the first year of the illness and affect up to 95% of persons diagnosed with dementia within the fifth year of the illness. (1) The BPSD are also the most important predictor of quality of life in the person living with dementia, of caregiver burden, and of placement in nursing homes or other long term residential care facilities—in turn a proxy predictor of survival in persons with dementia (8).

Even further removed from the conceptualization of dementia, is an understanding of the personhood, identity and autonomy of the person living with dementia. (7) Despite the incorporation of the four principles of medical ethics–beneficence, non-maleficence, respect for autonomy and justice into clinical practice and research; and the growing emphasis on patient centered rather than disease centered management; an appreciation of the identity of person with dementia is often lacking. (9–11) The person, so to speak, recedes behind the disease. However, it is this erosion of personhood, identity, and by extension, autonomy, that persons living with or at risk dementia and their caregivers most often express fear of. As noted by Bianca Brijnath, and Cohen, the person with dementia experiences death twice, once in the loss of identity; and then again at the end of life (7, 12).

## Identity, End of Life Care and Physician Assisted Death

The question of personhood, identity and autonomy, as well as of death, and the multiple forms it takes in persons living with dementia and their caregivers becomes even more relevant in a discussion of patient rights. (13) This includes, but is not limited to the right to live vs. right to die discourse often invoked in dementia (14).

The deterioration in cognition, emotion, behavior and functioning that occurs, in what is noted by the ICD-10 as a syndrome, usually of a chronic or progressive nature, is associated with an erosion of personhood and identity, and by extension, autonomy and competence in decision making. (15) The loss of identity—of a shared self, is recognized by some sociologists, including Brijnath and Cohen as a living death, or the first of the two deaths the person with dementia and their family must undergo (7, 12).

Thus, there is increasingly a need for shared (and assisted) decision making, advance directives (either care directive or a proxy directive or both). These provisions allow for and attempt to preserve patient autonomy for as long as is possible. (16) Yet, they often remain underutilized services that are not easily accessible to patients and families by virtue of incomplete penetration in the community, scarcity of opportunities and resources, and social inequity. (16) These services become increasingly relevant with the progression of dementia, loss of capacity and around the period of delivery of end of life care (13).

The National Cancer Institute (NCI), part of the National Institute of Health (NHI) defines end of life care as care given to people who are near the end of life and have stopped treatment to cure or control their disease. This includes physical, emotional, social, and spiritual support for patients and their families. (17) The end of life care is broadly divided into the following three, overlapping categories:

1. Palliative Care
2. Supportive Care
3. Hospice Care.

End of life care, including palliative care is therefore most often performed by physicians and care teams to the corpus of the person with dementia, based upon what is considered to be best practice and therefore in the best interests of the person. The caregiver, formal or informal, often has varying degrees of influence in the decision making process; depending upon the clinical context, level of risk associated with the decision or intervention, ethos of the dementia care team, legal provisions, and social (including individual and family) mores. Difficult decisions that are engaged with, though not often taken, during end of life care include prolongation of life, withdrawal of life support and assisted death (17).

Physician assisted death is defined by the Hastings Center as referring to the practice where a physician provides a potentially lethal medication to a terminally ill, suffering patient at their request they can take (or not) at a time of his own choosing to end his life. (18) The American Medical Association, in their Code of Medical Ethics Opinion 5.7 state that physician-assisted suicide occurs when a physician facilitates a patient's death by providing the necessary means and/or information to enable the patient to perform the life-ending act (e.g., the physician provides sleeping pills and information about the lethal dose, while aware that the patient may commit suicide). (19) The NCI goes on to define euthanasia (colloquially, though inaccurately known as mercy killing) as an easy or painless death, or the intentional ending of the life of a person suffering from an incurable or painful disease at his or her request. (20) Euthanasia by nature, as is widely known, can be active or passive (20).

The potential stakeholders in physician assisted death in persons with dementia may include:

1. Physicians
2. Nurses
3. Mental health professionals
4. Case workers
5. Patients
6. Caregivers (formal and informal)
7. Legal Representatives
8. Political and Legislative Representatives
9. Advocacy Groups
10. Professional Bodies.

While all the various stakeholders (formal and informal) in physician assisted death in general, and physician assisted death in persons in dementia in particular, are divided on the ethicality, morality, and legality of the same, this had been covered reasonably well in literature (14). Of these stakeholders, however, the least autonomy (and associated decision making capacity) often rests with the person with dementia, sometimes contemporarous with late stage dementia and end of life care (11). The potential for erosion of autonomy may indeed be more so in dementia than other terminal illnesses due to the inevitable decline of decision making capacity with the progression of the disorder (15).

This article intends to examine the thesis of how questions about the personhood, identity and autonomy of the individual may impinge on physician assisted death in persons with dementia. The approach is meant to place the identity of the person with dementia at the center of the discussion rather than take sides in the ever evolving right to live vs. right to die debate.

## The Self

At the core of personhood, identity, autonomy which play a role in guiding and shaping end of life care and the debate on physician assisted death is the self. The self can be loosely defined as an integration of the ideas of who we are, our existence in relation to other people, and a sense of worth and meaning derived from all of these (21).

At the core of an appreciation of the self in ourselves and in others, therefore, is the ability to realize the existence of self and delineate the self from the other or the environment. While the Cartesian duality of the mind and body may be artificial, an appreciation of the self is also associated with an anchoring of the self in the mind and the body. *I am myself, and not this person or that. I occupy this body and mind*.

This must be accompanied by the ability to localize the self in time and space, while paradoxically also maintaining a sense of continuity of the self over time and space. *I am 60 years old and am currently standing at this place. I am not who I was at 20 or who I will be at 80. Yet, I am a person who has lived through and experienced being 20 and may live through and experience being 80. I am at my house and not outside it, yet if I leave my house and go outside, I will still be myself*.

Further, this must be associated with a sense of perception, processing and praxis as the individual navigates their life in relation to others and the environment over time and space. *I can smell sense smells coming from the garden, which I associate with flowers and grass, and which are pleasant. By this, I realize that spring is here and I shall go the window or to the door to drink in the smells and sights of the garden*.

While the interaction with others and the environment occurs, there is also, constantly, a monitoring of and evaluation of the self. This appraisal of the self may be experienced in as a summing of strengths and weaknesses, of successes and failures, of mastery and pleasure, of what is in one's control and what is not, and of what one has achieved and of what is yet left undone. *I have worked and rested. I have loved and hated. I have raised children and grandchildren. I have been happy and sad. I have fulfilled these of my responsibilities but not those. I have experienced these difficulties and those pleasures. I have done this and there is that yet to do*.

Finally, there is a sense of meaning and of purpose, which emate from all the above layered appreciations of the self. *My life served this purpose. I had this meaning to my journey. I had this meaning to my life and that meaning to my death. I will be remembered for a while and by some after I die. I may eventually fade from memory, as all people do*.

These five concepts of the self are integrated into a whole, with a sense of oneness over time and space despite the changes accrued, and of ownership of the whole of the self. Personhood is a recognition that this self exists. Identity is a recognition of the contents of the self—of the whole and of its parts. Autonomy is governing over the self and the decisions that must be taken regarding the self. Agency stems from autonomy and is the ability to take action and experience outcomes based upon those decisions.

## The Self in Dementia

As would be clear from the above discussion of the components of the self and of its relationship to personhood, identity, autonomy and agency—these are complex cognitive function based abilities. While a large part of such an appreciation of the self-occur reflexively and often outside awareness, these nevertheless require a continuous utilization of cognitive resources. These cognitive resources also track the emotions, behavior and biological functions that the self-experience and constantly process and respond to them (15).

Dementia, with its progressive deterioration of cognitive function, in most cases, depletes the cognitive resources required for the integration of ideas that constitute the self. The impairment in memory and other cognitive abilities—amnesia, agnosia, apraxia, aphasia, alexia, agraphia, acalcuila, and executive dysfunction—are therefore not just primary losses of specific abilities of the brain, in and of themselves. They also cause a secondary inability to appreciate or integrate the self. *I used to be able to read the newspaper and remember the names of those in my family and purchase groceries. If I can no longer do this, whom am I?*

Persons with dementia differ in the level of insight and in the presence or absence of anosognosia, though decision making capacity declines with the progression of the illness (22). There is usually, however, some degree of distress about the loss of abilities and the changes in emotion, behavior and biological functions. *If I can no longer do this, what good am I to others? I was never this prone to losing my temper before, what has happened to me? Why do I now do this and not that?*

## Who is Alice? Does Alice Live Here Anymore?

The radical discontinuity over time and space that occurs in dementia then raises the question of whether the person now living with (and by extension dying with) dementia is the same person or someone else. Families and caregivers often articulate this when they report that they no longer recognize the person they are living with. While Brijnath and Cohen call this the first of the two deaths or the living death, Winston Chiong articulates this as the *someone else* problem, from the point of view of the other (13).

This postulation of living death has been contested. The slow decline in dementias, even when rapidly progressive, has no definite demarcations as are observed between life and death. It is therefore, unclear when exactly personhood undergoes sufficient erosion so as to constitute a loss of selves or a discontinuity in identity. Further, the living death is used in some countries, such as India, to indicate brain death. This paper uses the term in the common language sense (life emptied of joys and satisfactions in Merriam-Webster; an extremely poor quality of life, for example when someone is so ill that they are unlikely to recover in Collins) rather than in its narrower medical definition (23, 24).

*Alice is a woman in her seventies. Alice was once healthy. Alice now has dementia. Alice cannot now remember events that occurred in the past. Alice also cannot now form or retain new memories. Alice also finds it difficult to recognize people close to her or maintain her relationships with them. Alice once drove a car and balanced her check book. Alice cannot no longer do these activities. Alice had no fondness for sweets before developing dementia, but now has a marked sweet tooth. Alice can no longer regulate her bowel or bladder as she could do before*.

*Who then, is Alice? Is Alice then, still Alice now? Does Alice live in this body anymore?*

The continuity of self and of identity over time and space has been conceptualized as a question of:

1. Psychological continuity or continuity of the mind, vs.
2. Biological continuity of continuity of the body

To this may be added

3. Social continuity or a continuity of roles and relationships in society
4. Ethical and moral continuity or a continuity of values and beliefs held by the person.

Rebecca Dressler argues that the psychological discontinuity (loss of self) that occurs in persons with dementia, despite the biological continuity (habitation of the same corpus) implies that the person who existed before the occurrence of dementia, is not the same person who now lives with dementia (25). An alternate viewpoint, by Ronald Dworkin, has been that although there is a loss of self and erosion of identity, since the person still inhabits the same body, they are to be considered continuous (26). Therapies such as reminiscence therapy and narrative therapy tap into this discussion when they attempt to maintain psychological continuity over the lifespan of the individual by constructing a narrative out of shared memories between the person with dementia and caregivers (27). Medicines prescribed to the person with dementia also, by attempting to defer the decline in functioning, or minimizing the BPSD associated with the dementia, similarly attempt to maintain psychological continuity (28).

Further complexities are added by the change in the nature of relationships with dementia, from bilateral to inevitably unilateral, with disease progression (13). If the person with dementia in Alzheimer's disease can no longer fulfill the roles of a teacher or a parent or friend, are they still the same person they once were? Similarly, there often occurs a change in values and belief systems with cognitive decline and personality change. If the person with dementia in Pick's disease now believes physical aggression is acceptable in order to get one's way or if the person with dementia in Parkinson's disease now believes there is nothing wrong with gambling at cards or shopping excessively, are they still the same person they once were?

*How many changes must Alice accumulate in body and mind, until she stops being Alice? Does Alice cease to exist when she loses her abilities to think and relate and express or when her body dies?*

## How Discontinuity in Self and Identity Affects Personal Decision Making Capacity and Legal Competence

In situations where the person with dementia's personal decision making capacity is compromised by virtue of the illness and there is the possibility of legal incompetence, written advance care directives (living will), and/or nominated representatives, where available, are invoked. The preference, in medical care and in legal purview, is for shared decision making, and for a supporting patient autonomy and agency for as long as is safe and feasible. The two types of autonomy available to the patient in such a situation are (16):

1. Contemporaneous autonomy: This is the ability to utilize the self and identity to take a decision at the time (concurrent) when such a decision is required, such as whether to consent to undergo a surgery or to execute a deed of transfer of assets
2. Prospective autonomy: This is the utilization of information currently available in the public domain about the person's previous sense of (retrospective) self and identity, which are used to take a decision in the current context (prospective), such as whether based on previously held beliefs and values, the person would want to have now opted for prolongation of life.

There are two forms of prospective autonomy that are available to the person with dementia are for medical decision making are (16):

1. Documentary Directives: Written documents that contain instructions on how the person would (positive directives) and would not (negative directives) want to be treated
2. Proxy Directives: The appointment of a state sanctioned nominated representative (partial surrender of autonomy) or legal guardian (complete surrender of autonomy) who guides decision making based upon their conceptualization of the person's prior self and identity.

Both shared decision making and proxy decision making imply that there is sufficient discontinuity over time and space, by virtue of the dementia, to compromise the person's decision making ability in the specific context that is currently being invoked. Further, since current autonomy is compromised, previous autonomy is used as a substitute. *Alice can no longer express an opinion on whether she wants to undergo a joint replacement surgery for her arthritis or not. Yet, enough is known of the person Alice once was, and of the values and beliefs she held dear, to know that she would want to experience a comfortable and pain free life for as long as possible. Thus the clinician discusses with her family and decides to go ahead with the surgery*.

Yet, both also assume, that there is sufficient continuity over time and space that the person's core values and beliefs remain intact and do not alter as a consequence of the dementia. Where the person behaves in a manner contrary to previously held values and beliefs, such as in aggression or other harm-invoking behaviors, such is attributed to the disease rather than the person. *Alice enjoyed life and wished to lie a long, healthy and comfortable life. Now, there are occasions where she expressed, in bursts of anger that she would be better off dead and sometimes tries to hurt herself. When she says this, the clinician decides that these are part of the emotional and behavioral symptoms of dementia rather than an expression of the desire for a physician assisted death, and treats this accordingly with medicines and behavioral management*.

The expression of or demonstration of self harm is often transient and resolves with appropriate addressal and management of biopsychosocial factors as well as appropriate pharmacological management. Further, such agitation is usually indicative of an impairment of capacity to a sufficient degree so as to render the expressed desire to die invalid; though there is theoretical scope to interpret this in the context of previously enacted prospective autonomy. It is nevertheless, an indicator of a significant deterioration in subjective quality of life and merits examination of the goals of palliative care.

The discussion of continuity of identity becomes of particular importance where the current wishes of the person with dementia diverge from their previously expressed wishes. This is particularly so in end of life care and physician assisted death where the right to live vs. the right to die debate is invoked (29). In countries and states where the right to die in the form of the expression of directives for do-not-resuscitate (DNR) and/or euthanasia are not yet legalized, the clinician may be spared this potential conflict. Similarly, in countries and states where only negative, but not positive advance care directives are recognized, the burden of responsibility upon the physician is relatively less (30).

## Discontinuity and Physician Assisted Death

However, the discussion on self and identity ceases to be merely intellectual and enters the clinical context when physician assisted death is accorded legal sanctity. A dilemma may arise in the case of a person living with dementia, who had previously articulated a request for physician assisted death (in this case, euthanasia) while retaining decision making capacity. They now express a desire to continue life or deny making the previous request while decision making capacity is currently compromised by virtue of the dementia (13).

In delirium or functional mental illnesses, decision making capacity is assumed to be only temporarily compromised by the illness and treatment would ideally, restore the person to this capacity. However, the disintegration of self and discontinuity in time and space are far more lasting in persons with dementia.

In such cases, are the person's current wishes or previous ones more valid? Further, if there is a gross enough psychological discontinuity, do the advance directives now accurately reflect the wishes of the person living now, who clearly wishes to live?

Applying Dressler's argument, the discontinuity is sufficient that the current wishes of the individual be honored and given more merit than the previous directives enacted by the person who used to be prior to the dementia (25). The alternate argument, proposed by Dworkin, however, implies bodily continuity is sufficient to give precedence to the advance care directives over the current wishes of the person (26). Thus, ironically, identity becomes most important when considering the person with dementia, when it is least apparent, and mired by the disease.

The reverse scenario though, holds equally important dilemmas. Consider the person with dementia who had indicated through advance care directives that they would want to continue with end of life care dementia while retaining capacity, but now expresses a pervasive desire to die, while in compromised capacity. Do the latter instructions merit dismissal and treatment as symptom, by virtue of the illness?

*If we no longer know who Alice is or whether Alice lives here anymore, can we then, make out with any degree of conviction whether Alice would have chosen to live or to die?*

## Identity, Choices and the Dynamic Nature of Consent

The interaction between identity, dementia and end of life care (or where legal, the possibility of physician assisted death) may appear impenetrable, and a consideration of the same at length, pedantic. However, the intent behind such a deconstruction is to demonstrate the dynamic and ever changing nature of personhood, identity and autonomy in persons living with dementia.

As the person with dementia navigates the disease and deals with the progressive loss of abilities, disintegration of the self, and loss of decision making capacity—shared decision making and advance care directives, where available, offer support and the prospect of prospective or precedent autonomy. However, these options are best approached as dynamic processes rather than static events. There must be scope for the directives and decisions made by persons with dementia to change over time and space, in order to better reflect who they are at present (13).

Multiple recursive discussions may often be required, between the person with dementia, the clinical care team and caregivers (formal and informal) to evaluate capacity in each clinical context (31). This is best combined with repeated visitations of the person's advance care directives and proxy representatives, in a manner free from undue influence. This is important despite the asymmetry of power inherent to the clinical relationship (16). Where possible, it would be preferable to map the changes that may occur in values and belief systems due to discontinuity in identity over space and time (32).

Attempts must be made to understand and integrate these changes, where possible and to choose which of the decisions made by the person over time best represent their current identity and best interests. Advance care directives must be interactive and bear the potential for evolution.

## Fixed vs. Flexible Directives

Mental Healthcare Acts across most countries indicate the presumption of capacity unless proven otherwise. The Mental Capacity Act, 2005 in the United Kingdom, in addition to the presumption of capacity goes on to require that all practicable steps be taken to help persons make decisions and that they not be treated as incompetent merely because they make an unwise decision. It goes on to require that, in the absence of capacity, decisions made for persons be in their best interests and in the least restrictive manner. Similar civil safeguards are laid down in the Mental Health Care Act, 2017, in India.

Healthcare ethics also require that a clear clinical indication for evaluation of capacity be present and that an assessment be considered only when the behavior or decision to be made has significant consequences.

While guidelines for the evaluation of contemporaneous autonomy are clear, they are less so for when the creation of prospective autonomy must be considered. Both forms supplement each other and inform the clinical decision making process. The Study to Understand Prognoses and Preferences for Outcomes and Risks of Treatment (SUPPORT) indicates that the awareness of and utilization of advance directives and proxy representatives is low in persons with terminal illness and merely enhancing patient-physician interactions fails to address this deficit (33).

The author suggests that a discussion of prospective autonomy be included in the clinical interview of persons with dementia and their caregivers (formal and informal). Personal choices and preferences defining the terms on which people with dementia wish to live and die must be elicited during patient consultations, in a sensitive and meaningful manner. Physicians often wait for persons with dementia and their families to bring up the question of capacity (and autonomy), often in the context of a crisis such as the person being unable to live independently or manage their finances. This may mean that prospective autonomy is offered after, and not before capacity is compromised.

A pre-emptive discussion of advance directives and proxy representatives may enable collaborative decision making. Persons with dementia may require repeated discussions to facilitate education of rights and utilization of prospective autonomy. An improvement in the uptake of prospective autonomy may help patients navigate the degenerative process better. Inputs from the physician (such as the knowledge that negative, but not positive directives are legally binding) may also help persons with dementia draft and frame directives better.

Once established, it is also recommended to revisit care directives at periodic intervals, across the discontinuities in personhood, to ensure that prospective autonomy (once it takes effect) best represents the interests and wishes of the person they are now. There is merit in fixed intervals (such as 6 monthly or annual) for revisitation of prospective (but not contemporaneous) autonomy as well to elicit choices regarding palliative and end of life care. However, flexible revisitations, based upon clinical judgement and tailored to the individual may be more representative in person centered care. Further, the manner of framing by the physician may matter with experimental studies showing that expressed wishes for future care differ significantly between deficit focused and resource focused educational models (34).

## Indicators for Evaluation of Continuity

Potential indicators which may merit an evaluation of continuity of personhood and autonomy are:

1. Disease based—Transition in stages of cognitive decline (based upon instruments such as the Clinical Dementia Rating Scale) or changes in personality (as may occur in frontotemporal lobar degeneration)
2. Decision based—Such as when the person is required to make a treatment choice or decide upon care arrangements
3. Person based—When there is an indication that the person's outlook on life has changed significantly. This would include the disability paradox—since people often adapt to and find meaning in living with chronic illnesses that they may have once thought intolerable.

## Need for Instruments

There is a paucity of instruments to assess the continuity of personhood and identity across the spectrum of cognitive decline in older adults. Tools which may be useful in supplementing the clinical interview are (35, 36):

1. Identity Based: Personality Inventory; Present Behavioral Examination
2. Outlook Based: Philadelphia Geriatric Center Morale Scale; Measurement of Morale in the Elderly
3. Awareness Based: Knowledge of Memory Aging Questionnaire; INSIGHT
4. Capacity Based: McArthur Competency Assessment Tool; CURVES Capacity Assessment Tool.

## Conclusion and Future Directions

Much of the work on personhood and continuity of identity in persons with dementia remains theoretical. Clinical research, especially longitudinal studies examining the evolution of the sense of self, personhood, identity, capacity and choices would provide further insights. Another area of interest would be examining how attitudes toward dementia change with the availability of treatments which may modify or reverse the course of illness, such as the recently approved but controversial Aducanumab (37).

Where possible, end of life decisions must proceed with the consent or assent of the individual with dementia. Requests for physician assisted death, where legal, must be considered carefully, and at length, bearing in mind the potential for changes in identity and choice over the course of the illness. The living (and dying) will, if it is to fulfill its purpose and represent the person with dementia, should be truly *living*.

## Author Contributions

The author confirms being the sole contributor of this work and has approved it for publication.

## Conflict of Interest

The author declares that the research was conducted in the absence of any commercial or financial relationships that could be construed as a potential conflict of interest.
